# Quantitation of the cellular content of saliva and buccal swab samples

**DOI:** 10.1038/s41598-018-25311-0

**Published:** 2018-05-02

**Authors:** Christiane Theda, Seo Hye Hwang, Anna Czajko, Yuk Jing Loke, Pamela Leong, Jeffrey M. Craig

**Affiliations:** 1 The Royal Women’s Hospital, Parkville, Victoria, Australia; 2 Murdoch Children’s Research Institute, Parkville, Victoria, Australia; 3 Department of Obstetrics and Gynaecology, University of Melbourne, Parkville, Victoria, Australia; 4 Department of Pathology, University of Melbourne, Parkville, Victoria, Australia; 5 Department of Paediatrics, University of Melbourne, Royal Children’s Hospital, Victoria, Australia; 6 Centre for Molecular and Medical Research, School of Medicine, Deakin University, Geelong, Victoria, Australia

## Abstract

Buccal swabs and saliva are the two most common oral sampling methods used for medical research. Often, these samples are used interchangeably, despite previous evidence that both contain buccal cells and blood leukocytes in different proportions. For some research, such as epigenetic studies, the cell types contributing to the analysis are highly relevant. We collected such samples from twelve children and twenty adults and, using Papanicolaou staining, measured the proportions of epithelial cells and leukocytes through microscopy. To our knowledge, no studies have compared cellular heterogeneity in buccal swab and saliva samples from adults *and* children. We confirmed that buccal swabs contained a higher proportion of epithelial cells than saliva and that children have a greater proportion of such cells in saliva compared to adults. At this level of resolution, buccal swabs and saliva contained similar epithelial cell subtypes. Gingivitis in children was associated with a higher proportion of leukocytes in saliva samples but not in buccal swabs. Compared to more detailed and costly methods such as flow cytometry or deconvolution methods used in epigenomic analysis, the procedure described here can serve as a simple and low-cost method to characterize buccal and saliva samples. Microscopy provides a low-cost tool to alert researchers to the presence of oral inflammation which may affect a subset of their samples. This knowledge might be highly relevant to their specific research questions, may assist with sample selection and thus might be crucial information despite the ability of data deconvolution methods to correct for cellular heterogeneity.

## Introduction

The oral cavity is an excellent source of easy-to-access biological material for studies of genetics, genotoxicity, epigenetics, proteomics, metabolomics, and microbiomes^1–9^. This is due to the quick, non-invasive and low cost collection compared to tissues such as blood^9,10^.

The most popular sources of oral samples are saliva samples (collected by passive drool or swab) and buccal samples (collected by swabs or brushes). Both leukocytes (white blood cells) of mesodermal origin and squamous epithelial cells of ectodermal origin are found in the oral cavity (reviewed in^11^).

The epithelial cells present in oral samples can be differentiated into three cell types according to their characteristics following Papanicolaou (Pap) staining^12^. Basal and intermediate squamous cells have a blue cytoplasm, and after further differentiation to the superficial layer, cells stain pink in non-keratinous surfaces such as the inner cheek, and orange in keratinous surfaces such as the gingiva. Leukocytes, which are much smaller than epithelial cells, show the same morphological and stain characteristics as in blood smears and include mature granulocytes (segmented cells) and lymphocytes^11^. Little is known about the factors that influence the relative number of epithelial cells and leukocytes in the oral cavity, although one study found that in adults the presence of gingivitis was associated with a 34% increase in salivary leukocyte content^13^.

While cellular heterogeneity might have no impact on genetic sequence within an individual, with the possible exception of recombination in T and B lymphocytes, there are a number of reasons why knowing the proportions of leukocytes and buccal epithelial cells in oral samples is important. In adults with bone marrow allografts, for example, the proportion of blood-derived donor genetic material was shown to vary greatly; from 5–63% (median 21%) in buccal swabs and from 16–95% (median 74%) in saliva^14^. Therefore, genotype may vary in both saliva and buccal swabs following bone marrow allografts. A similar problem involving chimerism has been seen in fraternal twins that have shared the same placenta, an uncommon, but not rare occurrence^15,16^. More common reasons for knowing cellular content is for studies of epigenomics^9,17–19^, gene expression^20,21^ and proteomics^2^, in which the levels of analytes originate from mixed cell types, confounding case-control analysis. Although methods have been developed to adjust for cellular heterogeneity in epigenomic studies (reviewed in^17–19,22^), a knowledge of cellular heterogeneity in the primary samples would allow for more accurate adjustment^23^.

A simple, cost-effective and reliable method to screen saliva and buccal samples for their cell composition could provide researchers with important information (such as the presence of oral inflammation) which maybe relevant to their research questions and affect sample selection. Methods such as flow-cytometry (the “gold standard”) or statistical deconvolution methods allow for determination and correction of cell heterogeneity, but they can only be applied after more costly analyses have been performed.

To our knowledge, assessment of cellular content of oral samples from a wide age range of individuals (including children) by microscopy has not yet been performed. We therefore measured the proportion of specific leukocytes and epithelial cell types in saliva and buccal samples in children and adults. In children, we also tested the hypothesis that gingivitis, a common form of oral inflammation, has a major influence on blood cell content.

## Results

### Sample quality, slide staining and practical aspects of microscopy

Twenty child participants (i.e. ten twin pairs) recruited as part of the current wave of the longitudinal PETS cohort^24^ provided saliva samples and buccal swabs. Buccal swab slides were analysable from all twenty children (mean age 6.7 years, SD 0.2 years, range 6.4–7.1 years, 35% female) and saliva slides were analysable from sixteen children (same age distribution, 20% female). Of the twelve adult volunteer participants, buccal swab slides were analysable from eleven (mean age 36.3 years, SD 13.8 years, range 20–59 years, 82% female) and saliva samples from eight (mean age 42.6 years, SD 12.6 years, range 25–59 years, 75% female). Ages of the two sets of adults were not significantly different (*P* = 0.373). Slides excluded had none or too few cells on the slide with insufficient total cell numbers to perform the microscopic analysis.

**Figure 1:**
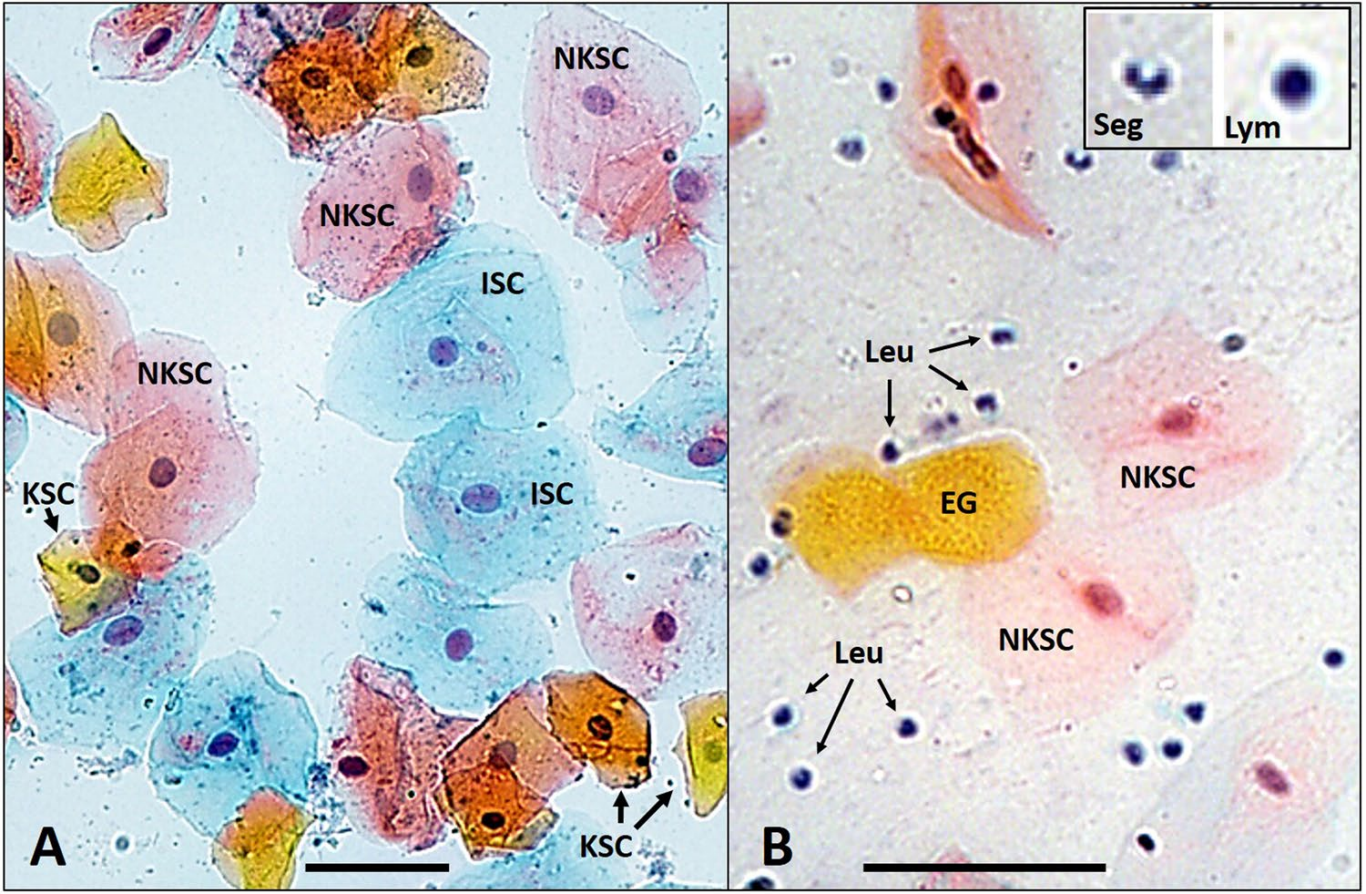
Examples of cellular morphology. Representative fields of view (200x magnification) of Pap staining of (**A**) a child’s buccal swab and (**B**) an adult’s saliva sample. The child’s buccal swab contains blue intermediate squamous cells (ISC), pink non-keratinized superficial squamous cells (NKSC) and orange keratinized squamous cells (KSC). The adult’s saliva sample also shows examples of enucleated orange ghost cells (EG), which were not included in our analysis, and a large number of leukocytes(Leu) with the insert showing a segmented cell (Seg) and a lymphocyte (Lym). The scale bars are 50 μm.

Representative fields of view from microscopy are shown in Fig. 1. There was slight variation in stain intensity, but pink, orange, and blue cells could be well differentiated. Epithelial cells were large with low nuclear:cytoplasmic ratios (Fig. 1A and 1B) and leukocytes were much markedly smaller with high nuclear:cytoplasmic ratios (Fig. 1B). Mature granulocytes were identified by the typical segmented appearance of their nuclei (thus also referred to as segmented cells) and lymphocytes were identified by their characteristically round nuclei surrounded by only a rim of cytoplasm (Fig. 1B). All other leukocytes were classified as “other mononuclear cells” and would be expected to include immature granulocytes, eosinophils and basophils.

Cell density per field was variable, and on average 6.3 (range 2–20) fields were reviewed to achieve the desired count of at least 100 total cells with at least 50 epithelial cells. Inter-observer variability in cell count and characterisation were not formally assessed but a >10% difference between the two observers (data discarded, see Materials and Methods) occurred for only 5 of 413 fields (1.2%).

### Epithelial cell to leukocyte ratios of saliva versus buccal samples

**Figure 2:**
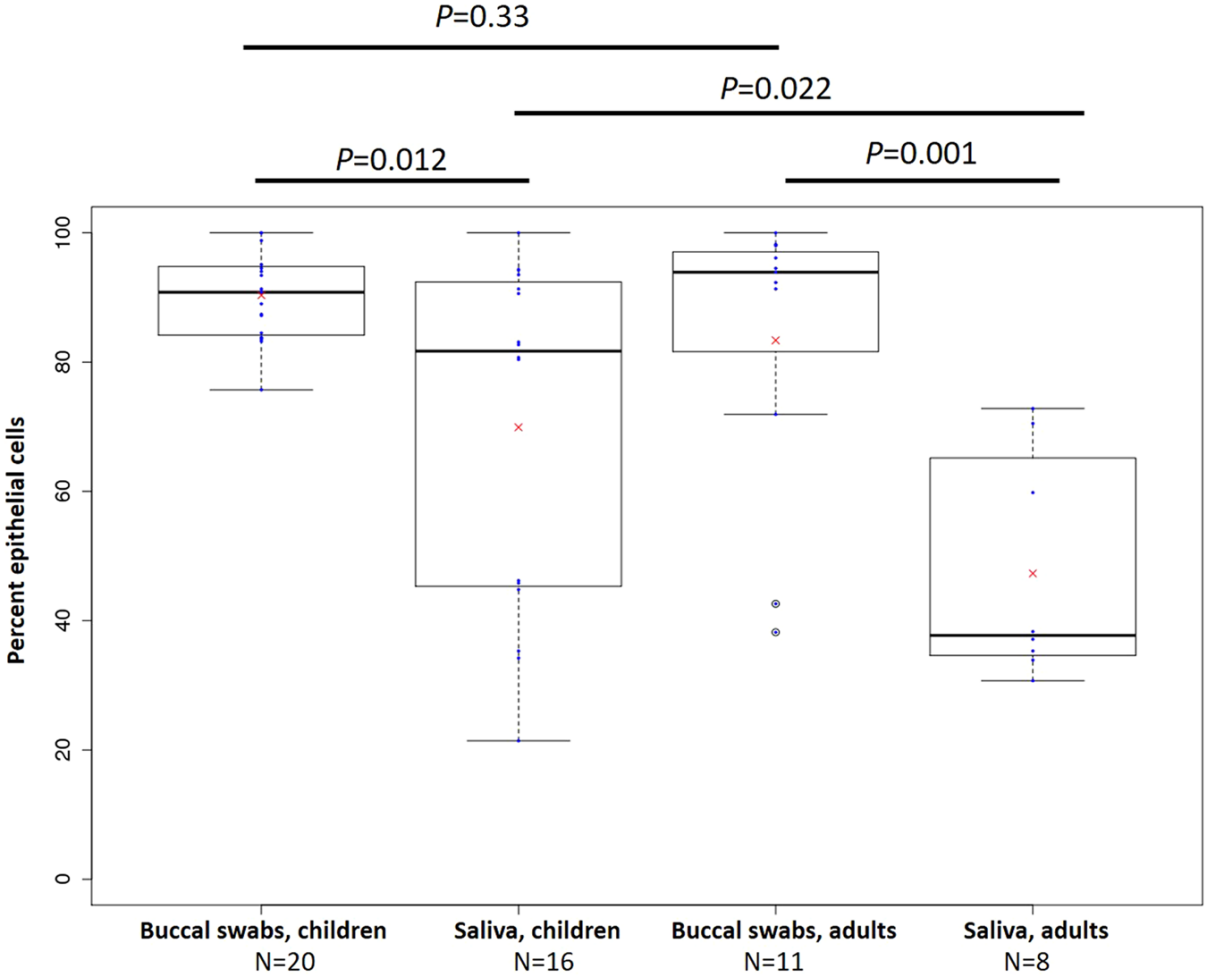
Box and whisker plots of proportions of buccal epithelial cells in buccal swabs and saliva from children and adults. Numbers of participants in each category are indicated and means are indicated with red crosses.

In the first stage of our analysis we determined the proportion of epithelial cells versus leukocytes, comparing buccal swabs and saliva samples from children and adults (Fig. 2). Both children and adults had significantly more epithelial cells in buccal swabs than in saliva samples. In children, the mean proportion of buccal cells in cheek swabs was 90.3% (SEM 1.5%) compared to 70.3% in saliva (SEM 6.8%), *P* = 0.012. In adults, the mean proportion of buccal epithelial cells in cheek swabs was 83.4% (SEM 6.8%) compared to 47.3% in saliva (SEM 6.2%), *P* = 0.001. However, between-subject variation, expressed as interquartile range (IQR), was much higher in saliva compared to buccal swabs in children (IQR = 46.3% and 10.3%, respectively) and adults (IQR = 27.5% and 15.4% respectively) (Fig. 2).

Although children had on average 7% more buccal epithelial cells in their buccal swabs than adults, this difference was not significant (*P* = 0.33). However, the proportion of epithelial cells in saliva was significantly higher in children than adults (+23%, *P* = 0.022), as was the between-subject variation (IQR = 46.3% and 27.5% respectively). We found no evidence of a sex effect in any analysis (*P* > 0.05).

### Specific buccal cell and leukocyte types

In the second stage of our analysis, we determined the proportions of specific cellular subtypes within buccal swabs and saliva samples. As previously mentioned, we could easily identify the three buccal epithelial cell types of intermediate squamous cells (blue), non-keratinous (pink) and keratinous (orange) superficial squamous cells. Nuclear:cytoplasmic ratios were similar in all cells and no basal or parabasal cells, which are characterized by a much smaller nuclear:cytoplasmic ratios^12^ were observed on any slides. Pap stain and morphological characteristics of the nuclei enabled us to differentiate leukocytes into segmented cells (mature granulocytes), lymphocytes and ‘other mononuclear cells’.

**Figure 3:**
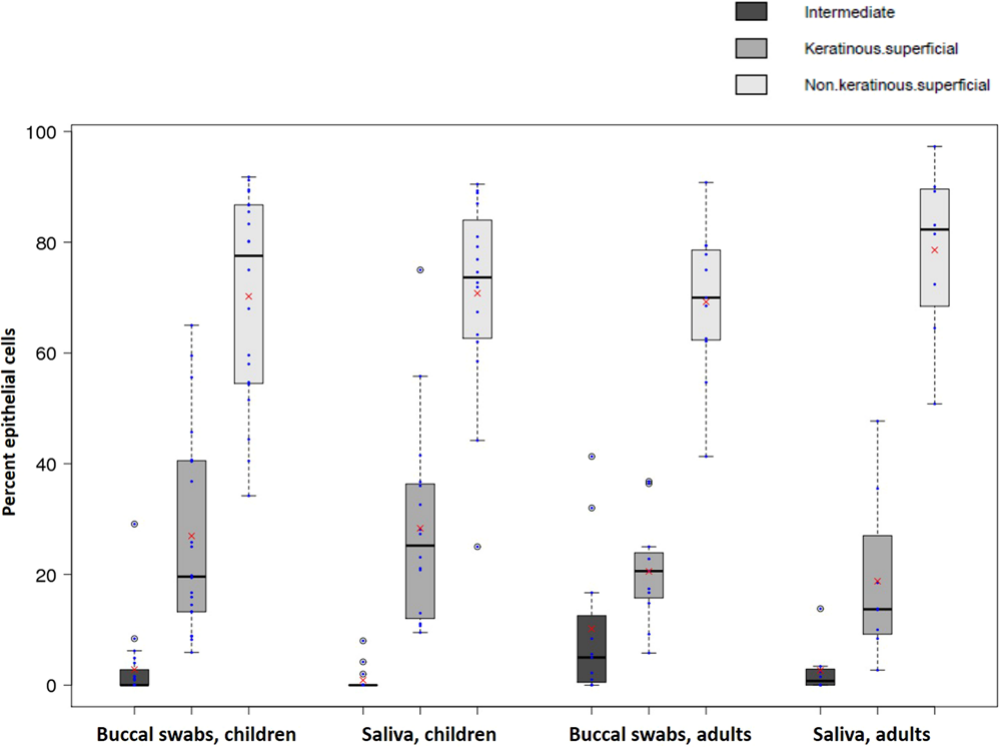
Box and whisker plots of three types of epithelial cells in buccal swabs and saliva from children and adults. Proportions of intermediate, keratinous and non-keratinous superficial cells are shown and indicated with different shades of grey. Means are indicated with red crosses.

**Figure 4:**
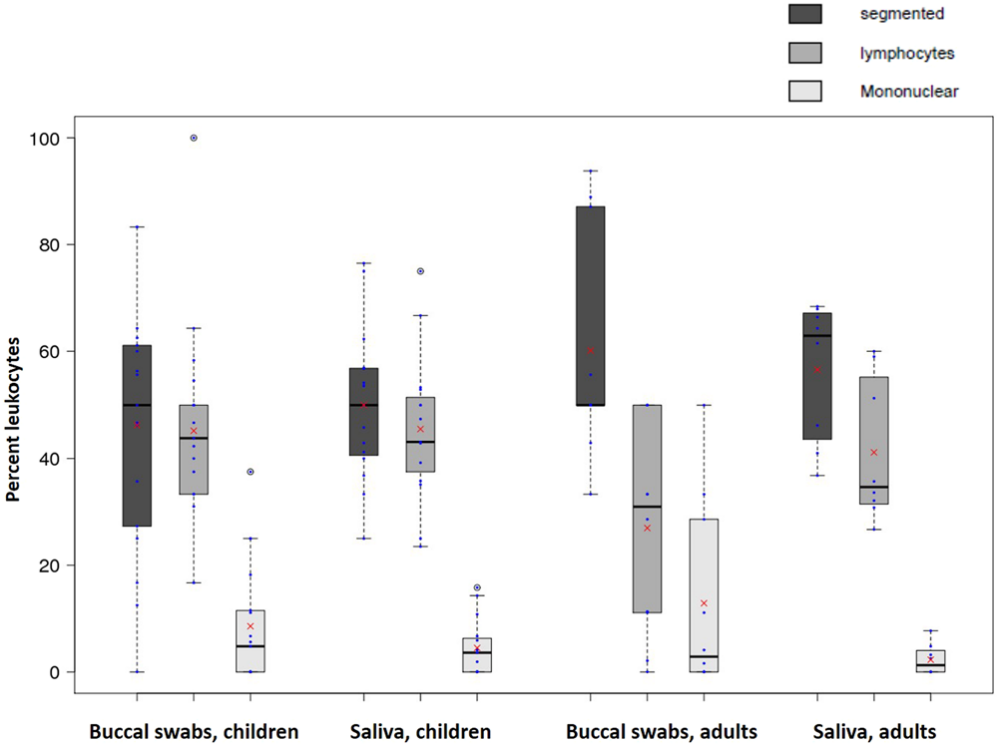
Box and whisker plots of leukocyte cell types in buccal swabs and saliva from children and adults. Proportions of segmented, cells, lymphocytes and other mononuclear cells are shown and means are indicated with red crosses.

By far the most frequent buccal cell type in cheek swabs were the pink non-keratinous superficial squamous cells (mean 70.5% of epithelial cells in children and 73.9% in adults), followed by the orange keratinous superficial squamous cells (mean 27.6% of epithelial cells in children and 19.6% in adults) and the blue intermediate squamous cells (mean 1.9% of epithelial cells in children and 6.4% in adults) (Fig. 3). We did not include the orange ghost cells into our analysis as they do not contain a nucleus and thus are not relevant for DNA based studies, which are our main interest. The most frequent leukocytes were segmented cells (mean 48.1% of leukocytes in children and 58.4% in adults), followed by lymphocytes (mean 45.4% of leukocytes in children and 34.1% in adults) and other mononuclear cells (mean 6.9% of blood cells in children and 7.6% in adults) (Fig. 4).

### Does gingivitis influence leukocyte counts

**Figure 5:**
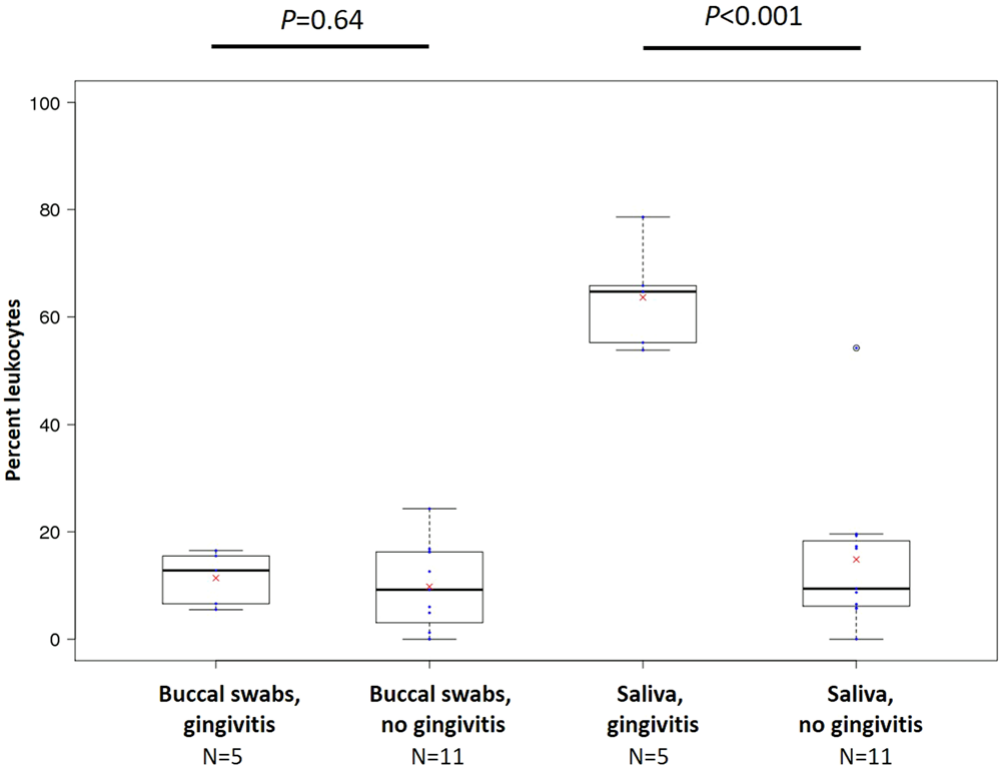
Box and whisker plots of the presence of leukocytes in saliva and buccal swabs in relation to gingivitis. Numbers of participants in each category are indicated means are indicated with red crosses.

In the third stage of our analysis, we tested our hypothesis that the presence of gingivitis would be associated with a higher proportion of leukocytes in buccal swabs and saliva samples. We had comprehensive data on oral health for our child participants only. We found no evidence for an effect of gingivitis on leukocyte content in buccal swabs: 11.0% of cells in those with gingivitis were leukocytes compared to 8.9% in those without (*P* = 0.638). However, we found that the levels of leukocytes in saliva were almost 50% higher in children with gingivitis (63.6% of all cells) compared to those without (14.9% of all cells, *P* = 7.2 × 10^−6^) (Fig. 5).

## Discussion

Saliva and buccal samples are increasingly being used for medical research studies including those using modern “omics” platforms. As outlined in the Introduction, for some studies the mix of buccal epithelial cells and leukocytes can be highly relevant. Moreover, these two cell types are of different developmental origin as buccal epithelial cells are ectodermal and leukocytes are mesodermal in origin. This is especially relevant for studies evaluating the role of DNA methylation and other epigenetic marks in human health and disease; it has been shown that due to their ectodermal origin, buccal epithelial cells are a better proxy for the brain than peripheral blood^18,25^. Cellular heterogeneity has been shown to be a major confounder in such studies^26,27^. We therefore set out to establish the cellular content of two commonly collected oral samples – buccal swabs and saliva samples. To our knowledge an assessment of cellular components of buccal and saliva samples by microscopy has not yet been done for children and adults.

With regards to microscope slide preparation, we had greater success analysing buccal swabs than saliva samples as a greater proportion of saliva samples had insufficient cell quantities on the microscopy slides. While not specifically addressed in our study, issues of cell quantity issues could be addressed by increasing the cell density of saliva samples by centrifugation and resuspension in a smaller-than-original volume of PBS.

We found that both sample types contained a mixture of epithelial cells and leukocytes. This is not surprising because the buccal mucosa is relatively permeable and has a rich blood supply^28^. We also found that the epithelial cell content of buccal swabs was significantly higher than saliva in children (mean 90.3% in buccal swabs, 69.9% in saliva) and adults (mean 83.4% in buccal swabs, 47.3% in saliva) (Fig. 1). The most likely reason is that the direct scraping of the cheek enriches for such cells, whereas saliva will only contain sloughed-off cells. Buccal cell proportions in adults were similar to that observed in a study that used genotyping of allograft recipients of a similar age (78% in buccal swabs, 31.1% in saliva)^14^ and that derived from flow-sorting (42.2% in saliva)^13^. Differences between studies could be due to methodological issues including the use of flocked swabs (our study) compared to cytology brushes^14^. We also found that the between-subject variation in epithelial cell content was much higher in saliva samples compared to buccal swabs in children and adults. This could relate to variation in the presence and/or severity of oral inflammation (see below for further discussion). Taken together, these results suggest that buccal swabs are preferable to saliva for maximising epithelial cell content of oral samples. Cellular heterogeneity is important to take into account in a wide range of downstream applications^2,9,14,16,19–21^. Therefore, we recommend that buccal swabs are preferred over saliva samples for studies of cellular function and that cell counts are performed where practical, especially where there are no established methods for post-hoc adjustment for cellular heterogeneity. If time and materials are limited, data can be adjusted for cellular heterogeneity post-hoc using reference values, as it has been done for studies of DNA methylation (reviewed in^22,23^).

We found that the mean proportion of epithelial cells in saliva was 23% higher in children than adults. Assuming that the age does not affect the quantity of epithelial cells collected from an individual, adults could have more blood cells present in saliva due to the higher prevalence of periodontal diseases. Chronic periodontitis, for example, is the most common form of periodontal disease affecting approximately one third of the adult population and is not usually found in the mouths of children. It is a major cause of tooth loss after the age of 25 years^29^.

Using Pap staining we could differentiate between three types of nucleated buccal endothelial cells. The most numerous cell types were pink-stained non-keratinous superficial cells, followed by orange keratinous superficial cells. The least numerous of cell types in all samples from adults and children were intermediate endothelial cells, which lie just beneath the surface of the oral epithelium. These cells were slightly more abundant in buccal swabs from both children and adults; this might be due to the direct scraping of the mucosal surface with the swab. Overall, the epithelial cell content in buccal swabs and saliva is relatively homogeneous. However, as there are likely to be some functional differences in the three cell types observed, this could confound some downstream analyses, although nowhere near as much as for leukocytes, which come from a completely different cell lineage.

Healthy buccal mucosa contains a number of cells involved in immune function including lymphocytes (including T cells), segmented cells (polymorphonuclear, including neutrophils and eosinophils) and mast cells^11,28,30^. We are aware that our differentiation of leukocytes in this study is an estimate only as we did not apply any specific stains which allow for better differentiation of leukocyte types nor did we use a magnification high enough to perform a detailed leukocyte analysis. We could confirm though, that the most common types of leukocytes in blood - lymphocytes and segmented cells - were also the dominant leukocytes in buccal and saliva samples from both children and adults (Fig. 4). We found no major differences in the proportions of these cells between groups, indicating that cells of both the innate and adaptive immune system are universally present in the oral cavity. In blood, segmented cells are twice as numerous on average as lymphocytes and around ten times as numerous as other mononuclear cells. Our results indicate that there might be an enrichment for lymphocytes in oral samples but this would have to be confirmed with better cell type differentiation. Without further functional characterisation it will be impossible to conclude whether such cells migrated to the oral cavity passively or actively, although evidence from other studies points to the latter^28^.

We found that the presence of gingivitis in children was associated with a ~50% (4.3-fold) greater salivary leukocyte content (Fig. 5). This agrees with a previous study of adults (mean age 33 years) which found 1.3-fold greater number of salivary leukocytes using flow cytometry^13^ and suggests a correlation between oral inflammation and saliva leukocyte content. As gingivitis was associated with differences in cell composition, visual oral assessments in children may be particularly important to identify other sources of inflammation such as oral trauma, ulcers or cheek biting. We are unsure why buccal swabs from children did not also show a greater leukocyte content in the presence of gingivitis. One possible reason is that rinsing the mouth and/or collection of saliva prior to cheek swabbing removes the majority of leukocytes originating from the gingival surface.

Strengths of this study include the wide age range of participants, the identification of multiple types of buccal cells and leukocytes and the inclusion of data on oral infection in children. Weaknesses include the small sample size (data from 20 children and 11 adults), cell differentiation count by non-specific stain and observer assessment only, rather than use of more specific stains or the “gold-standard” method of flow cytometry. We did not quantify total cell numbers (not feasible in buccal swab samples) and we were not able to further investigate and validate our results through application of post-hoc statistical deconvolution methods following epigenome-wide array analysis^25,31,32^. We also lack data on oral inflammatory lesions in adults.

In conclusion, we found that saliva and buccal swab samples almost always contain a mix of leukocytes and epithelial cell in a wide range of proportions, especially in saliva. We recommend that buccal swab sampling should be the method of choice to reduce cellular heterogeneity for downstream studies. Researchers could perform a quick oral examination or could ask research participants about oral inflammation including gingivitis, periodontitis or ulcers, at the time of sample collection. However, our method provides a low-cost tool to alert researchers and verify the presence of oral inflammation which may affect a subset of their samples, especially in children. This knowledge may be relevant to specific research questions, may assist with sample selection and thus might be crucial information to obtain before performing more involved and costly analyses despite the availability of deconvolution methods to correct for the cell heterogeneity.

## Methods

### Participants

Child participants included ten pairs of twins from the Peri/postnatal Epigenetic Twin Study (PETS) cohort, an Australian twin birth research study based in Melbourne^24^. Adult samples were collected from twelve volunteers at the Murdoch Children’s Research Institute. Each participant or their legal guardian provided informed consent and provided one saliva and one buccal sample. Ethics approval was obtained through the Human Research Ethics Committee of the Royal Children’s Hospital, Melbourne (project no. 33174) and all methods were performed in accordance with the relevant guidelines and regulations.

### Saliva samples

Saliva samples were obtained first. Participants were advised to consume nothing but water within 30 minutes before sample collection and ten minutes prior to sample collection, to rinse their mouths with water. Saliva was collected unstimulated via passive drool for 3–5 minutes to collect at least 1 ml. One hundred microlitres of saliva were applied to a microscope slide, smeared and immediately fixed with 95% ethanol for 10 minutes and left to dry at room temperature.

### Buccal swabs

After saliva collection was completed, buccal swabs were collected with two Copan FLOQSwabs, Interpath, Heidelberg West, Australia), using methods that involved a member of the research team rubbing swabs up and down against the inside of each participant’s check twenty times, then in the maxillary and mandibular buccal sulcus (the upper and lower furrows between the gingiva and the inner cheek) for ten seconds per side. Each buccal swab was wiped along the length of a standard size microscope slide and fixed as outlined above for saliva.

### Oral Assessment

Child participants underwent an oral examination by a trained dental examiner to determine whether there was any inflammation or infections that may affect the cell counts or diversity in the samples obtained. The presence of any oral lesions, signs of inflammation or infections, and tooth decay was recorded. For the adult participants a brief oral assessment by a trained dental examiner was conducted. As for the child participants, this was to determine and record the presence of any inflammation, infections, oral lesions or evidence of past or present tooth decay.

### Slide staining and microscopy

Slides were stained using a routine Papanicolaou (Pap) stain^33^ at the Royal Children’s Hospital Pathology Department. All slides were deidentified and analysed by two observers simultaneously using a dual microscope setup. Light microscopy was performed at a 200x magnification. The two observers (CT and SHH) counted cells (as below) for each field of good quality, i.e. adequate stain, and appropriate cell density to enable cell counts. Counts were compared and used if discrepancy between observers was less than 10% of total count. If discrepancies were over 10%, a new field was chosen to count. The observers moved through each slide until at least 50 epithelial cells and a total of at least 100 cells minimum from at least two fields of view had been counted. Epithelial cells were differentiated as cells with (1) blue, (2) orange or (3) pink cytoplasm; enucleated orange-staining ‘ghosts’ were also noted. Leukocytes were differentiated into (1) segmented cells, (2) lymphocytes or (3) other mononuclear leukocytes.

### Data analysis

Cell counts for each field were noted during the microscopy sessions until the running total reached the cell number criteria (as outlined under above). Data were then entered into Microsoft Excel for analysis. Box and whisker plots show the following: boxes, interquartile ranges (IQR, 25th to 75th percentile); solid horizontal lines within the boxes, medians; whiskers, data from 5th to 95th percentiles; blue dots, raw values; white circles, outliers; red crosses, means.

### Data availability

Our raw data is freely available.
